# “We Thought We Were Alone”: The Subjective Experience of the Siblings of Anorexic Adolescent Patients

**DOI:** 10.3389/fpsyt.2021.664517

**Published:** 2021-05-17

**Authors:** Angelo Persico, Salome Grandclerc, Catherine Giraud, Marie Rose Moro, Corinne Blanchet

**Affiliations:** ^1^APHP, Hôpital Cochin, Maison de Solenn, Paris, France; ^2^CESP, Fac. de médecine – Univ. Paris Sud, Fac. de médecine – UVSQ, INSERM ≪ DevPsy ≫, Université Paris Saclay, Villejuif, France; ^3^Université de Paris, PCPP, Boulogne-Billancourt, France

**Keywords:** anorexia nervosa, siblings, mental representations, support group, group program, family approach, qualitative research

## Abstract

**Objective:** The siblings of patients suffering from Anorexia Nervosa (AN) are potentially affected by a disturbed emotional experience that often remains undetected. In order to bring them a psychological support, the Maison de Solenn proposed a support group program for these siblings. The current research explores their mental representations of AN and their emotional experience in the support group named “sibling group.”

**Method:** This exploratory study is based on a phenomenological and inductive qualitative method. Four girls and three boys aged between 6 and 19 participating in the “sibling group” were included in a one-time focus group session using a semi-structured interview guide. The thematic data analysis was performed by applying the methods of interpretative phenomenological analysis.

**Results:** Themes that emerged from the interview fall into four categories: AN explained by siblings; the individual emotional experience of siblings; the family experience of siblings and the experience inside the “sibling group.”

**Discussion:** According to our participants, the “sibling group” thus functions as a good compromise between keeping an active role in the anorexic patient's care and taking a step back to avoid being eaten up by the illness. Sibling-group participants retrieved a sense of belonging, which is normally one of the functions of being a sibling. It is important to note that the “sibling group” is part of the comprehensive (or global) family-based approach included in an institutional multidisciplinary integrative care framework.

## Introduction

Anorexia Nervosa (AN) is a psychiatric pathology characterised by food restriction behaviours, leading to insufficient body weight, intense fear of getting fat, and changes in the perception of body image. Epidemiological studies have shown that AN is more frequent among girls aged 15–19 with a prevalence rate of 0.3% and an incidence of 8 cases per 100 000 per year in the general population (1–4).

This disorder often starts during adolescence, with prolonged evolution and a non-negligible risk of chronicity. Besides individual somatic and psychiatric complications, which can lead to life-threatening situations, there are also major psychosocial and familial repercussions that lead to a deterioration of the patient's and the family's quality of life (5–7).

Numerous reports and a wide body of research have shown that chronic pathologies have deleterious repercussions on family functioning and on each one of its members, including siblings, who are constantly concerned by the upheavals linked to the occurrence of a chronic illness (8–14). In the case of AN, professionals have in the last few years developed family-centred approaches, providing care to anorexic children or teenagers alongside support for the parents, but neglecting care toward the brothers and sisters, whose emotional experiences have long been ignored (15–18). This exclusion is also reflected in the medical and psychological literature, where there is very little information or research devoted to the impact of AN on patients' siblings (19–25).

In the first studies that focused on assessments of siblings' personal experiences, brothers and sisters expressed themselves along these lines: “knowing what's going on, being a little more active, it would have helped me not to feel so left out” or: “as for me, I'm transparent, apparently not interesting.” The comments that were collected from the brothers and sisters of anorexic patients in a study on support for families of teenagers with an eating disorder clearly showed their feelings of exclusion and powerlessness.

A study of siblings' coping strategies for dealing with their sister's AN reported 22 different coping strategies, including fourteen emotion-focused, five problem-focused, and three support-seeking strategies. Among the emotion-focused strategies, “empathising with my sister, forgiving her behaviour,” “distancing from negative affect” and “developing cognitive reconstructions, making meaning,” were some of the emerging themes (20–23, 26, 27).

Siblings can also experience feelings of guilt, worry, fear, and pain, but also difficulties in tolerating separation on the occasion of hospitalisation of a brother or sister. These emotions are often hidden and experienced alone by siblings, who do not take it upon themselves to ask for help from their family (20, 23). On the other hand, they may feel obliged to take care of their anorexic brother or sister and to give their parents emotional support; paradoxically, so as to avoid any conflict, they avoid telling their parents about any pathological behaviour they have witnessed (20, 21, 28).

A recent meta-synthesis of qualitative studies concerning the experience of “caring for or living with someone with an eating disorder” reported that siblings describe AN as having a pervasive and all-encompassing impact on their daily lives. There is some evidence that this may affect their mood, identity, body perception, attitudes to food, family relationships, home life, school functioning, and motivation. Siblings feel obligated to care for their ill sibling and support their parents, who ignore their concerns about eating disorders (29).

This distressing and potentially psychologically harmful personal experience generally goes unnoticed; brothers and sisters find themselves powerless to cope with the incomprehension surrounding the illness (23, 30).

Because of the clear impact AN has on the dynamics of inter-sibling relationships and on their psychological state, a support group or “sibling group” was set up in the adolescent multidisciplinary department of the “Maison des Adolescents—Maison de Solenn” (31), to address the need to support the siblings of anorexic patients. The objective is to let them express themselves and give them the support they need in their personal experience, which can be overwhelming, painful and incomprehensible to them.

This initiative was started at the behest of some parents who had taken part in a parental exchange group at the time they were confronted with their child's anorexia, a discussion group created in 2005 at the “Maison des Adolescents—Maison de Solenn.” Parents reported on the siblings' worries concerning their brother's or sister's state of health, and the feeling that they were being left out and excluded from any form of care. Furthermore, these parents were worried about these other children, their well-being and their development when faced with the family upheaval necessarily triggered by the illness. Moreover, they themselves felt caught up in their child's illness and felt they were not available enough to provide support.

This “sibling group” is based on different studies that have been conducted on the need and the advantages of including siblings in the care provision for patients with chronic somatic or psychiatric pathologies (8, 12). These studies are all the more relevant in the field of eating disorders because the occurrence of such disorder changes and questions family functioning. Family relationships are in many authors' view paramount to understanding AN, whether for its genesis, its maintenance or its treatment (30). There is a need to pay attention to family bonds, which are seriously undermined by the illness, through regular family interviews, and in some cases family therapies.

Providing care and trying to relieve these siblings' painful experiences is one of the first aspects of the support group program that is presented here. But this group also has a place in the overall therapeutic process of the family approach, which designates the family as a therapeutic resource to combat the illness (32–35).

The “sibling group” meets on a monthly basis for 1-h sessions. It is led by a doctor expert in eating disorders and adolescent medicine, a child psychiatrist and a clinical psychologist. The group is open to anyone and the number of participants may vary, as sessions are not compulsory.

The sessions are structured with a first part devoted to free talk, where no subject is imposed. The participants are invited to suggest a topic freely, so that shared reflection can emerge from the group. This joint reflection leads the participants toward a common elaboration of their experiences of having an anorexic brother or sister. In a second part, a ludic support is suggested to help the participants to express themselves. Professionals offer adolescents different types of mediation including role playing, drawing, writing, photolangage. Indeed, the use of mediating objects can help to shape subjective experiences and supports the psychic process of subjectivation (36).

In this research, one of the issues concerned the ideas, feelings and perceptions the group participants had about anorexia. We also sought to cast light on their personal emotional experiences, as well as their degree of motivation for taking part in the group sessions, the advantages felt by each of them and their assessment of the ludic supports.

To address these various questions, it was decided to run the research with two main objectives:

- To explore representations the sibling-group participants had of AN.
- To assess the personal emotional experiences concerning the “sibling group” allowing feedback from the siblings on the proposed group therapy system in order to improve our practises.

## Materials and Methods

This study was observational and exploratory, based on an inductive, phenomenological, and qualitative approach. A qualitative methodology was chosen since it helps to understand subjective perceptions, and the significance and the meaning of a phenomenon on the basis of detailed narrative reports (37, 38). A qualitative methodology was particularly suited to the complexity of the study subject. A phenomenological approach was thought to be the best adapted to study experiences of suffering (39, 40). It also took the subjective and constructivist aspects into account, where knowledge is the fruit of a shared construction between the researcher and the participant (41–43). This method thus enabled the participants to take on the role of experts on their own personal experience (44). Concerning the qualitative methodologies, we have chosen to refer to a method of discourse analysis: the Interpretative Phenomenological Analysis (IPA) technique, which will be presented in more detail below.

### Sampling

The sampling was reasoned and the selected subjects represented typical cases (45). The subjects were recruited among sibling-group participants between 2017 and 2019. They were therefore brothers and sisters of all ages of adolescents with AN who were currently or had been treated at the “Maison des Adolescents—Maison de Solenn” (Cochin Hospital, Paris, France). As the sibling group is an open group, siblings may have regular or occasional participation over a period of time. This disparity in the regularity of participation in the support group reflects the heterogeneity of the sibling group in clinical practise, so we decided to include in the research siblings who had participated in the sibling group even for a short period.

### Data Collection

For the data collection, a socio-demographic datasheet was used, as well as a one-time focus group session structured by an interview guide and two ludic supports.

#### Socio-Demographic Datasheet

The socio-demographic datasheet collected information on age, gender, address, school level, rank among the siblings, family composition, date of entry in the “sibling group” and the number of participations at sibling group sessions.

#### Focus Group

This technique of group interviews allows a structured exploration of the participants' different points of view on a subject. It is based on group dynamics and provides opportunities to collect experiences, needs, expectations, and representations. These interactions and feed-backs encourage the emergence of knowledge, opinions and experiences as a chain reaction as a result of the meeting of a wide range of personalities which encourages the expression and discussion of controversial opinions. The researcher is like an explorer, he may know part of the subject but will also discover unknown areas. Methodological thoroughness is essential as much for the data collection as for its analysis (46–49).

### Procedure

To organise a the focus group session, a semi-structured interview guide was created by the authors, defining two categories (Table 1):

**Table 1:** Thematic guide for the semi-structured interview during the focus group.

**1. Representation of the illness:** Report on the descriptive and conceptual aspect of the illness. • What do you think anorexia nervosa (AN) is? • How would you describe a person with anorexia? • What do you think people think about anorexia? **2. Emotional Experience:** Explore the emotions associated with ill siblings. • How do you feel when you think about your sibling? • What feelings would you like to express to your sibling? • Can you think of a scene with your sibling that was difficult for you? • Can you think of a scene with your sibling that was enjoyable for you? **3. Siblings' place in the family:** Describe siblings' experience based on their place in the family • How do you feel about your family? • How do your parents or other family members experience your sibling's anorexia? • Could you describe a family meal? • Could you tell us about a family scene that particularly affected you? • Are there any differences between your family and a family not affected by anorexia? **4. Impact on the adolescent process:** To explore the influence of the illness experience on sibling adolescence • Do you think your adolescence was influenced by your sibling's anorexia? Why or why not? • Do you think your adolescence would have been different if your sibling did not have anorexia? • Do you think that your brother's or sister's illness has influenced the way you are? **5. Free expression** • Are there any feelings that you experienced during this interview? • Is there anything you would like to add about your experience?

- Representations of anorexia: this aimed to give an account of the ideas and feelings concerning AN disease, the patient suffering from AN, the family and the experience of having brother or sister with AN. These representations were then assessed to see how they had changed over the course of subsequent sessions of the “sibling group.”

- The participants' emotional experiences in the “sibling group”: this aimed to explore the participants' subjective experiences in the group during the sessions, through the emergence of affects, the participants' level of motivation and their perceptions of the group's dynamic but also but also the opinion of brothers and sisters on the ludic tools being used during the support sibling group.

Two ludic group activities were offered to mobilise participants' emotional expression and symbolic abilities. These mediations helped the participants clarify their ideas of their own representations and to put them into words during the session:

- Emoji cards: a set of 25 cards representing emotional states in the “emoji” format. The instruction was to choose the cards that best represented the emotions they experienced during the sibling group sessions.

- A wish-list: participants were asked to draw up a list of ideas to improve the group session program. Participants were also invited to write down their wishes or suggestions for the following year in anonymous manner.

All the brothers and sisters who agreed to take part signed informed consent on a form explaining the objective and the nature of the study. Each participant's data was stored in a socio-demographic data file. The focus group took place during an hour-and-a-half group session in the “Maison des Adolescents—Maison de Solenn,” which was audio-recorded. It was conducted using the interview guide (Table 1), which was the main thread for the whole session. Three researchers (AP, SG, and CB) conducted the interview.

The framework and objectives of the focus group were clearly stated to the participants. We also explained that in order to guide our discussion, we would use an organised semi-structured interview guide and two ludic supports that were offered in the middle and at the end of the interviews. We made it clear that each participant was entitled to his or her own way of expressing feelings, points of view and personal experience. Finally, we insisted on the rules of respect for each other's opinions and confidentiality. All datasets (generated and analysed) for this study are included in the manuscript and the tables.

### Data Analysis

Data analysis was carried out according to the Interpretative Phenomenological Analysis method, which enables the study of the meanings subjects build from personal experiences (50–52). A meticulous analysis of the interviews, as they took place enabled a set of super-ordinate themes to emerge, which were themselves linked to several themes describing all the narrated experiences. The themes were not only chosen on the basis of their prevalence in the data. Other factors, including, in particular, relevant passages casting light on the themes, and the way different themes helped to shed light on other aspects of the narrative, were also taken into account.

Once a the focus group session was recorded, it was transcribed in full. The analysis of the material was obtained from meticulous scrutiny by three researchers (AP, CB, and SG) of the transcription content. First of all, each researcher independently identified themes that they considered best represented the content of the interview. Then the three researchers compared their results and agreed on a common analysis.

The methodological criteria were retrospectively verified according to the COREQ (COnsolidated criteria for Reporting Qualitative research) checklist (See Supplementary Material 1).

### Ethical Considerations

Participants in the “sibling group” were invited to take part in the study completely freely. The care provided to the anorexic brother or sister was not in any way conditioned by their participation in the research.

All the participants were informed of the research objectives in an informed consent form. For minor participants (under 18 years old), a consent form for minor participants allowing them to take part in a study was signed by the parents and their child/adolescent. In both documents, the framework and objectives of the study were clearly explained. All subjects (siblings and their parents) gave written informed consent for the research and for the publication of the datasets (social and demographic characteristics of the study population and direct quotes from the participants) in accordance with the Declaration of Helsinki.

The group interview was recorded with agreement by all participants. It was retained solely for the purpose of transcription. Once the material had been transcribed, the audio tape was destroyed. The resulting written material remained confidential and was used solely by the research team for content analysis. All interviews have been anonymised to get the datasets non identifiable.

This study was carried out in accordance with the recommendations of an appropriate ethics review board (“Comité de Protection des Personnes—Ile de France,” ref. CPP: 2016-A00818-43, 3426). The protocol was approved by the ethics review board “Comité de Protection des Personnes—Ile de France.”

## Results

### Population

The participants in the research included 4 girls and 3 boys who had attended a sibling-group session at least once. Their ages ranged from 6 to 19. Their characteristics are summarised in Table 2. The participants' brothers or sisters were all treated at the Maison de Solenn.

**Table 2:** Characteristics of the study population.

**Participant**	**Brother 1**	**Brother 2**	**Brother 3**	**Sister 1**	**Sister 2**	**Sister 3**	**Sister 4**
Age	11	13	19	7	12	12	15
Group participation (months)	2	24	7	2	6	1	24

### Thematic Analysis

From analysis of the data with the IPA method, 4 meta-themes were suggested: AN as explained by the siblings, the siblings' individual emotional experiences, the siblings' family experiences and their experiences in the “sibling group.” These categories provided a way of organising, understanding and reporting the experiences that the participants were able to express during data collection. The first three meta-themes refer to the first objective of the study: exploring the siblings' representations of anorexia. The last meta-theme is related to the second objective, which aimed to evaluate the experience of the siblings within the proposed group setting: the “sibling group.”

#### AN Explained by Siblings

This category focused on the way brothers and sisters understood anorexia, on the basis of their ideas, perceptions, and hypotheses about the illness.

##### It Is Like Imprisonment

The siblings focused on the idea that the illness leads anorexic patients toward regularly withdraw, which excludes them from interpersonal contacts. This isolation prevents them from feeling happiness and vitality, thus leading them to a feeling of solitude.

*Brother N*°*1: “it's like being unable to feel anything that brings happiness… you don't see anyone anymore, you shut yourself up, inside your own bubble, it's like feeling that everybody's attacking you. As if you were truly alone…”*

*Brother N*°*3: “It'll be hard for them to keep their friends, they lock themselves up inside their own delusion, it just goes round in circles…”*

##### There Is a Need to Self-Harm

For siblings, anorexic children deliberately try to self-harm because they deserve it and it gives them a certain satisfaction.

*Sister N*°*4: “she did like - self-mutilation… (she) feels she deserves it, and so the fact of inflicting that sort of thing on yourself, it gives you a good feeling, in the sense that you deserve it… As if it was a punishment… because you need to harm yourself…”*

##### It Never Gets Completely Better

The siblings did not believe that a full recovery was possible in the end. For them, there would always be internal signs and after-effects from the illness.

*Sister N*°*4: “I don't know if you get cured from this illness… you can manage… well… to overcome things, and so on, but there's a special relationship with food. There will always be signs… more on the inside with the after-effects. Because on the outside, they can be wiped out if you want…”*

The siblings seemed to think that cure is synonymous with going back to a previous state.

*Brother N*°*1: “Well, there'll be problems, but there are problems more or less everywhere… There will always be memories of this time… you never get back to your previous state, maybe a little bit below that… Body-building, drawing… it makes you forget, but as soon as you sit down for a meal and you have to eat, doesn't it (the illness) come back to you?”*

#### The Siblings' Individual Emotional Experiences

This category gives an account of the brothers and sisters' emotional experiences associated with anorexia.

##### We Do Not Understand Why

The siblings expressed their difficulty in understanding the causes of the illness. Unlike other organic illnesses, there is no concrete explanation about its causes and no details on its treatment.

*Sister N*°*4: “We often ask ourselves “why”, but there is no particular reason. It's not like an illness where you've got this or that germ… It's complicated. There are many different factors…We try to understand, but we don't know anything, so we feel ill at ease.”*

##### We Are Powerless

Powerlessness is a feeling that comes from the inability to contribute to the cure of anorexic siblings, if only because of the restricted verbal communication. The participants felt that the support they had to offer did not have any real impact.

*Brother N*°*3: “In fact, what's important is to accept the fact that there are moments when there really is a sense of powerlessness.”*

*Brother N*°*2: “You can't do anything to cure them, apart from talking to them… we are powerless because we can't talk to them… you can't say to them “why can't you do it? What's the matter?””*

##### We Feel Angry

The siblings felt angry toward their anorexic brother or sister, as well as with themselves. Behind this feeling is the idea that anorexic adolescents are responsible for their illness.

*Brother N*°*3: “Feelings of powerlessness can sometimes turn into feelings of anger… We can also believe that they are responsible for what's happening to them… that they're doing it on purpose.”*

*Brother N*°*2: “You can't talk to them, you feel angry and sometimes you swear at them… You say that maybe it's their fault… Sometimes you get angry at yourself.”*

#### The Siblings' Family Experiences

This category concerns the way the siblings experienced the family upheavals triggered by the illness.

##### It Is Hard for the Parents

The siblings realised that the illness made things difficult for the parents, who first of all seemed to react through denial. The siblings pointed out that the parents' role was undermined by systematic, generalised refusal on the part of their anorexic child.

*Brother N*°*3: “We lie to ourselves. We say to ourselves “he's not anorexic”. The child finds it hard to accept, in fact… for the family, it's really hard to accept”*.

*Brother N*°*1: “it's really hard when he says “no, no” and he pushes people away…for example: “do you want to go for a walk?”… “No, I don't feel like it” and it's always the same… “Have you eaten your watermelon?”… “No, I haven't”… There it is… he refuses everything… they (the parents) are a little bit all over the place, they try to, they really do try to free the child from anorexia, to help the child. Er… and it's really hard because… the anorexic child refuses. So, they don't know how they can help…”*

##### We Try to Help

The siblings try to give a helping hand in the family, which potentially leads to ambivalent feelings, between gratification and suffering. To do this, they try to find certain strategies to prevent their brother or sister from getting angry or feeling guilty or in distress, or to avoid any conflict with them.

*Sister N*°*2: “We act as if it was our duty to help our sister… it makes us feel good inside, but at the same time, it destroys us…With my sister, you can never talk to her after dinner because she's inside her own bubble… she blames herself and she gets angry.”*

*Brother N*°*2: “We ask questions “how was your day?” just small talk… Then, we ask questions like “what's the matter with you?”… We try to make a little more progress, to have fewer taboo subjects, so as not to shock him of course.”*

*Brother N*°*1: “What I'd say to my anorexic sister if I hadn't spoken to her in a long time is “nice to see you”. But not “how are you” or anything like that. Because it might give her a shock”*

##### We Keep Our Distance

Finally, the siblings also keep their distance in order to deal with their own feelings and protect themselves.

*Brother N*°*3: “My sister (non-anorexic) was a little angry with my brother (anorexic) because she thought he was in a way responsible for what had happened to him… As for me, I tried to keep my distance.”*

##### We Have to Stay Out of It, Without Leaving Them Completely in the Dark

According to the siblings, anorexic children need the presence of their family, but at the same time need to keep their distance. Patients have to learn to become autonomous. However, this balance seems difficult to find. The siblings can feel they are abandoning their anorexic brother or sister.

*Brother N*°*3: “At the same time they (the patients) need a family presence… and they also have to fend for themselves. They have to break away… to feel they are independent in relation to the family.”*

*Brother N*°*1: “I agree, but not entirely, because you can't leave them in the dark… they are lost…that way.”*

#### Experience Inside the “Sibling Group”

This category gives an account of experiences of the “sibling group” participants during the session and their perceptions of this support group program.

##### We Were Able to Express Things

Participants felt supported by the other brothers and sisters who shared the same experiences. It enabled them to express themselves without being bothered by the judgements of other people with no experience of the illness, and without being afraid of hurting their parents when recounting their own experiences. It also enabled them to feel less alone.

*Brother N*° *3: “it helps to put thoughts that we usually keep to ourselves into words… it's a change from other people we meet… who lecture us … when they know absolutely nothing. I prefer to hear about other siblings' feelings… it's a lot more authentic. And in the end, I find it a lot more interesting…”*

*Brother N*°*2: At first we thought we were the only ones. But as soon as we were here, at the Maison de Solenn, we felt less lonely. We were able to talk about things.”*

*Sister N*°*3: “It's also about… being able to talk, in the end, without hurting other people. Like our parents.”*

##### Attending the Group Sessions Enables us to Keep Our Distance and Provide Help at the Same Time

Participation in the “sibling group” allowed the participants to feel less trapped in the family dynamic, whilst keeping an active role. It helped them understand and deal with the illness better.

*Sister N*°*4: “This is exactly why there are institutions like this one. So that we can get away from the family but continue to be able to help.”*

*Brother N*°*1: “At the beginning, I didn't know what to do… er… I knew nothing about the illness and then, er… well, I started to know things about what I should or shouldn't do.”*

*Sister N*°*4: “I don't think that it changed anything, but perhaps we understand things better… And then, we manage to deal with it better…”*

*Brother N*°*3: “I thought that my brother would be pleased to see that his brothers and sisters were also part of the Maison de Solenn in some way.”*

##### Fear, Sadness, and Difficulty Talking, but Also Happiness (Table 3)

Through the ludic supports used during the focus group, participants were able to express feelings that had not been mentioned previously. These feelings were fear, sadness, difficulty talking or listening to other siblings' experiences, but also feelings of relief, to be able to express themselves and the joy that their brother or sister was still alive (Table 3).

**Table 3:** Analysis of the ludic support: ≪ Set of Emoji cards ≫.

**Participant**	**Chosen cards**
Brother 1	1. Surprise: “He's surprised.” 2. Joy: “He feels ready and he doesn't feel bad. There's no real stress.” 3. Euphoria: “This is where I am, he's happy to go there… really happy.”
Sister 1	1. Falling asleep: “He's asleep.”
Brother 2	1. Panic: “He's desperate.” 2. Sadness: “Because he's desperate, he's crying” 3. Fear: “Because he's crying, he's afraid.” 4. Anger: “Because he's afraid, he's angry because there's nothing he can do.” 5. Joy: “But after that, he's ok…” He says he feels good even so… because she (the anorexic sister) is still here, isn't she?.”
Sister 2	1. Refusal to talk: “I prefer to listen to how the others are feeling.” 2. Sadness: “It makes me sad to see that we feel we're, er… in the same situation.” 3. Joy: “It's also good to talk.”
Sister 3	1. Refusal to listen: “I don't really want to hear because it makes me sad.” 2. Sadness: “I'm sad because we are talking, and because, well, my brother is still hospitalised.” 3. Anger: “It's a little embarrassing.” 4. Joy: “But I'm glad to be able to talk about it a little.”
Brother 3	1. Refusal to talk: “He doesn't want to talk about certain things. And I understand that.” 2. Anger: “During the last session, there was someone who was so rude, I thought it was a shame that he wasn't put back in his place. The session was completely ruined… What he had to say had nothing to do with the spirit of the group. I also thought he showed no respect for anyone.” 3. Joy: “He's smiling because the activity gave me confidence to be able to express myself… and in different ways.”
Sister 4	1. Refusal to talk: “We don't really know how to talk and we don't necessarily want to talk either.” 2. Panic: “We talk about things and we don't know how to react to these things…” 3. Refusal to listen: “I don't particularly want to hear things, sometimes it's a bit complicated…”

*Participants chose the Emoji cards that best represented their emotional state during the “sibling group” sessions. They then commented on their choice and gave more in-depth answers. Overall, the participants chose images representing the following states: surprise (1 participant), joy (5 participants), euphoria (1 participant), falling asleep (1 participant), panic (2 participants), sadness (3 participants), fear (1 participant), anger (3 participants), refusal to talk (3 participants), and refusal to listen (2 participants)*.

*Brother N*°*2: (he selected cards with expressions of panic, sadness, anger, fear and joy) “he's desperate, and because he's desperate, he's crying. Because he's crying, he's afraid. Because he's afraid, he's angry, because he can't do anything. But afterwards, he's all right. He says he feels good nonetheless… because he (the anorexic brother) is still here.”*

*Sister N*°*3: (She selected cards with expressions of disgust, sadness, anger and joy) “I don't really want to know, because it saddens me… I'm sad, because we're talking about it, because my brother, well, he's still hospitalised. It's a little embarrassing… but I'm glad we can talk about it a little.”*

##### We Want to Talk About Subjects That We May Not Have Thought About (Table 4)

Finally, the participants wanted to go deeper into subjects that had not been broached until then in the group. The experience of an outsider to the group could thus help to open up exchanges on this kind of theme. They seemed interested in an account given by an “expert” patient who had gone through the different phases of the illness (Table 4).

**Table 4:** List of wishes in the second ludic support.

- Suggest several activities for a session and let people choose. - Suggest a few sessions closer to one another. - Intervention from a former anorexic patient or an expert patient. - Explanation of certain aspects of the illness from the professionals' point of view. - Talk about more complex subjects. - Invite people to talk about their own experience if they want to.

*In this support the participants provided the following suggestions*.

*Brother N*°*3: “I would have liked to talk about more complex subjects that we might not have thought about… let's say, for instance that you… you talk about someone else's experience. These are subjects that we don't always think about…”*

## Discussion

### Siblings Take on the Role of “Translators” of the Illness

This exploratory study aimed to explore the representations that siblings of anorexic children had of AN and how far they were able to give form to these representations through their participation in the group, to know whether their participation enabled any psychic elaboration to take place. The second objective of this exploratory study was to assess the personal emotional experiences concerning the support group program.

First of all, we observed that the representation the participants had of AN was completely that of a psychological subjective experience. Indeed, the brothers and sisters explained AN in terms of imprisonment, isolation, absence of joy, distrust of the external world, and repetitive delusional behaviours. These representations showed that their approach was obviously more emotional than rational or theoretical. In other words, the siblings seemed to put themselves in their brother or sister's position to try and understand what they were going through from a relational and affective viewpoint (53).

We could thus suggest that the “sibling group” participants attempt to understand AN through their own interpretation and deduction of the emotional states of their suffering brother or sister. This interpretative approach of the siblings could be associated to the concept of mentalization whereby individuals are able to interpret their own's and other people' actions by basing themselves on mental states, such as personal desires, needs, feelings, beliefs and reasons (54). Siblings thus try to attribute mental and emotional states to the patient's pathological behaviours. It could provide them with some explanation of what anorexic symptoms can express through the attack on the body, the psyche and interpersonal relationships. We believe that this type of elaboration, is related to the fact that AN does not have a single identified aetiology unlike most somatic diseases and cannot be explained or treated like them.

However, this effort of mentalization could also be considered as an attempt to compensate the failure of the anorexic sibling's ability to mentalize. The lack of mentalization in anorexic patients has already been mentioned and explained by similar concepts like “reflexive function” or “social cognition.” According to these concepts, AN is associated with a dysfunction of the psychological processes underlying the construction of mental representations and to a poor emotional mental state inference. The representation ability of patients with AN would not be able to contain an experience. Instead, the representations are assimilated to “presentations,” which are experienced as concrete facts in the here and now. These “pseudo-representations” are described as “concretised metaphors” in which a feeling of control can refer to an empty stomach, or a firm, slender body can be equated with a feeling of purity, for example. Similarly, in terms of lack of social cognition, self-starvation and purging behaviour may serve as inappropriate strategies to cope with impaired recognition of other people' emotions (55–57).

Therefore, it could be said that siblings assume the role of “translators” of the disease, by attempting to put themselves in the place of the anorexic sibling, inferring their thoughts and emotions, building mental representations of their interpersonal relationships, giving deeper meaning to symptoms that would otherwise be not understandable and transforming a concretised mode of expression into a language of representation, full of meaning, emotions, and metaphors.

As an example, in this study the siblings used the metaphor “self-mutilation” to explain anorexic behaviour in terms of the need to self-harm. In this case they talked of mutilation as an analogy, which could at least partially explain the patient's fundamental motivations. They consider that anorexic patients seek to self-harm because they feel they deserve it and it somehow gives them pleasure.

This work of understanding of the psychic states could be also related to the Bion's notions of “reverie,” containment and the alpha function (58). This concept designates the mother's capacity to receive her small child's projective identifications, to use them as communication and to return them in a modified form. Thus, containment is the capacity to receive the unelaborated and rejected contents of someone else, to experience them, to transform them and to return them to the subject in a modified form with signification (59).

Making an analogy with this model, siblings attempt to metabolise anorexic patients' raw psychic contents (beta elements) through their own ability to daydream. This process consists in giving anorexic patients' behaviours and feelings meaning, in order to metabolise and transform them into thought activities (60). By giving anorexic symptoms meaning, the siblings change an invasive illness into an emotionally charged phenomenon for their anorexic brother or sister.

It was also noted that the siblings were unwilling to spontaneously share their representations of AN with their family. They feared that their way of interpreting the illness might be difficult to understand for those who were not in the same elaboration process. Consequently, the siblings felt that their family's judgemental reaction could be violent and sanctimonious. This fear is all the more intense when siblings imagine that the expression of their emotions could hurt their anorexic sibling and their parents. In this case, the feeling of guilt would make their own personal experiences even more painful.

This guilt may also be related to the desire of adopting a “care giver role” in the family. The persistent failure of sibling strategies to support and soothe the emotional state of the ill sibling and his or her parents may develop into a sense of self-blame, powerless and anger. Cook-Darzens (30) refers to this type of sibling as a “pseudo-parent” because they try to care for and protect the anorexic child. This role may be triggered by a desire to compensate for their parents' weaknesses and to inhibit their own aggressive tendencies.

When faced with this emotional experience, siblings could choose to distance themselves from their family in a radical way, in order to protect themselves from this painful and disturbing situation. Yet this distancing increases their feelings of guilt and worry concerning their sibling (22, 61). This crossroads leads to ambivalent feelings between remaining in close contact with anorexic siblings, thereby maintaining their role of translator and helper, or staying away from them and preserving themselves. The presence of ambivalent feelings can also be observed when the siblings express opposite feelings such as joy and sadness, or fear and relief.

Thus, the siblings seem to be at higher risk of finding themselves alone in a very demanding psychological elaboration process in relation to the brothers and sisters with AN, dealing with their own feelings of uncertainty, fear, guilt, anger, powerless and ambiguity, without the containing function of the family.

### The “Sibling Group:” a Good Compromise to Find the “Right Distance”

Our participants felt the need to find the “right distance,” allowing them to avoid any excessive contact with their anorexic brother or sister. That way, they had a sense of independence and at the same time provided their anorexic brother or sister with affective support and presence. This position enables siblings to protect themselves from excessive proximity without having the feeling of neglecting the anorexic sibling. On the basis of this notion of the “right distance,” we could hypothesise that siblings are conscious, to a certain extent, of the risks of the dynamics of a symbiotic relationship. Indeed, on the basis of what the siblings said, families should not function in an overprotective, idealised and intrusive way. It has been shown that this type of family functioning damages the process of identity development, in an attempt to protect families from conflict, aggression and traumatic events in their intergenerational storey (62).

Of course, this balance between presence and distance is not easy to reach. It requires elaboration, and challenges intra-family roles. The space where siblings can take a step back in relation to their family, whilst remaining faithful to their feelings, representations and behaviours within the family dynamics, might therefore be found in the “sibling group.”

According to our participants, the “sibling group” thus functions as a good compromise between keeping an active role in the anorexic patient's care and taking a step back to avoid being eaten up by the illness, which overturns intra-familial relationships. Inside the group, siblings find a place to express themselves outside the family on what is happening inside it. Their feelings of guilt are alleviated, which enables emotional expression, without any fear of hurting their parents or their anorexic brother or sister with bouts of anger, powerlessness or frustration.

Similarly, siblings seem to be supported by the idea that in the group “we are all living through the same thing,” which allows them to feel confident enough to share their representations. In this notion of shared experience, participants retrieved a sense of belonging, which is normally one of the functions of being a sibling (63), but which was undermined by the illness. Through the links woven between the participants in the “sibling group,” their lack of identification references in the family thus seemed to have found a way forward for elaboration.

Finally, it was confirmed that the introduction of playful ludic supports was preferable to an approach that was solely verbal in the “sibling group.” This allowed the siblings to set free their expressions and their emotions, even the ambivalent ones that oscillated between joy and sadness or between the need to talk and the refusal to talk. A solely verbal approach seemed to us insufficient to tackle this kind of emotional complexity, particularly when these emotions were re-enacted during the sessions.

### Study Limitations

One of the limitations of our study was the small number in the sample. A study with a larger number of participants could provide results that could be more readily generalised.

We are also aware that the differences in the participants' ages played an important role in the analysis of the results. Indeed, the understanding of the experience by a 6 year-old participant is different from that of a 19 year-old young adult. It is therefore difficult to talk about a completely homogenous experience for the group as a whole. It is important to remain cautious and take each participant's individuality into account.

One other aspect of this individuality was the variability in participation in the “sibling group,” which was an open group. Our study participants did not taken part in the group program with the same regular attendance nor from the same starting date.

Patient gender but also sister or brother gender could have an influence on the sibling experience of anorexia nervosa, particularly during the adolescent process where identity issues are very common. Only one of our patients suffering from anorexia nervosa was an adolescent boy. This is consistent with epidemiological data reporting a sex ratio of 1 male to 10 females (64), although the prevalence of eating disorders in boys may be underestimated.

Finally, the absence of a control group was a limitation in our study. It would have been interesting to compare the participants' experiences among our sibling-group subjects with the experiences of siblings who did not take part in this group, either by choice or because it was inaccessible, in order to observe the effects of the group program on the different types of representations of anorexia, the participants' abilities for psychic elaboration, their styles of emotional expression and their personal experiences concerning family relationships.

## Conclusions and Perspectives

From this research, we were able to observe that the “sibling group” allowed the participants to benefit from a remedial experience. Through the group dynamics, siblings found identification elements, enabling them to have a sense of belonging. This gave them enough confidence to express their anxieties, as well as their ambiguous and aggressive feelings.

We were able to observe the fact that the brothers and sisters were able to have enlightened opinions concerning the family dynamic. They were able to detect the need for the right distance between the members of the family, which made the relationships more balanced. It is important to be able to develop the siblings' therapeutic potential within their family. Their ability to elaborate and the richness of their personal experience could prove beneficial for the patient in a family therapy framework, or during regular family interviews. By providing some support for identification, by confronting similarities and differences and by making emotions communicable, the “sibling group” acts as a prop to the siblings' subjectivation. This process, as well as being individual, can also have an impact on the family, particularly on relationships. Through the group intermediary, siblings could enable the family system to open up to new information and multiple interactions (32).

It is also important to note the institutional aspect of the “sibling group.” This program is part of a wider care provision integrated into the family approach, including direct care of the anorexic patients and their parents. This institutional environment seems to provide a very important containing function for the siblings, who feel they are involved in an overall treatment program.

We can thus confirm that siblings of anorexic adolescent patients require specific care, taking account of their individual and subjective way of understanding the family, with their own ideas, analogies, and emotional elements. Care solely centred on information communicated by professionals in charge of patients with AN to the siblings could be seen as detached from the affective and idiosyncratic aspects. It would be good to communicate this approach to teams dealing with adolescents suffering from AN, and alongside to be able continuing this work with a wider study including a larger number of participants and a control group as previously mentioned.

## Data Availability Statement

The original contributions presented in the study are included in the article/Supplementary Material, further inquiries can be directed to the corresponding author/s.

## Ethics Statement

The studies involving human participants were reviewed and approved by Comité de Protection des Personnes - Ile de France III, réf. CPP: 2016-A00818-43, 3426. Written informed consent to participate in this study was provided by the participants' legal guardian/next of kin. Written informed consent was obtained from the individual(s), and minor(s)' legal guardian/next of kin, for the publication of any potentially identifiable images or data included in this article.

## Author Contributions

AP and CB contributed to conception and design of the study. AP, SG, and CB conducted the interview and analysed the interview to optimise the validity of the results. AP wrote the first draught of the manuscript. AP, SG, CB, and MM wrote sections of the manuscript. All authors contributed to manuscript revision and read and approved the submitted version.

## Conflict of Interest

The authors declare that the research was conducted in the absence of any commercial or financial relationships that could be construed as a potential conflict of interest.
